# Gentamicin-loaded exosomes from IMMUNEPOTENT CRP enhance healing of infected diabetic wound in mice

**DOI:** 10.3389/fphar.2025.1682468

**Published:** 2025-11-27

**Authors:** Paola Leonor García Coronado, Brandon Alberto Garza Martínez, Kenia Arisbe Moreno Amador, David Reding Hernández, Diana G. Zárate Triviño, Diana Elia Caballero Hernández, Pablo Zapata Benavides, Gabriel Luna Barcenas, Cristina Rodríguez‐Padilla, Moisés Armides Franco Molina

**Affiliations:** 1 Laboratorio de Inmunología y Virología, Facultad de Ciencias Biológicas, Universidad Autónoma de Nuevo León, San Nicolás de los Garza, Mexico; 2 School of Engineering and Sciences, The Institute of Advanced Materials for Sustainable Manufacturing, Tecnológico de Monterrey, Queretaro, Mexico

**Keywords:** IMMUNEPOTENT CRP, exosomes, wound healing, diabetic ulcer, gentamicin, regenerative

## Abstract

**Introduction:**

Diabetic foot infections (DFIs) are a major cause of lower extremity amputations and are associated with substantial morbidity and reduced quality of life. Given the limited efficacy of current treatments and the rise of antimicrobial resistance, there is an urgent need for innovative therapeutic approaches. This study evaluates the use of exosomes derived from a bovine leukocyte spleen extract (IMMUNEPOTENT CRP), loaded with gentamicin, to improve infection control and promote wound healing in a diabetic setting.

**Methods:**

The efficiency of gentamicin encapsulation were evaluated followed by gentamicin release under acidic and alkaline conditions. A wound model was established in streptozotocin (STZ)‐induced diabetic mice, followed by inoculation with *Staphylococcus aureus* to simulate infected diabetic ulcers. Mice were treated with gentamicin-loaded exosomes (Exo‐Genta), IMMUNEPOTENT CRP‐derived exosomes (ICRP-Exo), or free gentamicin. Wound closure was assessed for 21 days. On days 0, 7, 14, and 21 skin tissue samples were collected from treated mice to evaluate epithelial thickness, area, and cell number calculation by hematoxylin and eosin (H&E); collagen synthesis, and PI3K-AKT pathway activation, beside skin samples, blood samples were collected to quantify pro‐inflammatory cytokine levels.

**Results:**

The Exo‐Genta and IMMUNEPOTENT CRP significantly enhanced collagen fiber deposition, blood vessel formation, and hair follicle regeneration. At the molecular level, these treatments increased AKT phosphorylation and modulated the inflammatory response, with reduced levels of TNF‐α, IL‐6, and MCP‐1, alongside a significant increase in anti-inflammatory IL‐10.

**Conclusions:**

Gentamicin‐loaded exosomes derived from IMMUNEPOTENT CRP demonstrated enhanced antimicrobial activity and tissue regeneration in infected diabetic wounds. These findings support their potential as an effective and less invasive therapeutic strategy for diabetic foot ulcers by combining infection control and pro‐regenerative and immunomodulatory effects.

## 1 Introduction

Diabetes mellitus (DM) is a rapidly escalating global health concern, affecting an estimated 537 million adults in 2021, with projections rising to 643 million by 2030 (IFD, 2022). Among its complications, diabetic foot ulcers (DFUs) represent one of the most debilitating, affecting 15%–25% of patients during their lifetime, with infection occurring in nearly half of these cases (Armstrong et al., 2017). Diabetic foot infections (DFIs) are the leading cause of lower extremity amputations, resulting in substantial morbidity, disability, and impaired quality of life.

The economic burden of DFUs and DFIs is considerable. In the United States alone, annual costs for DFI management are estimated at $9–$13 billion (Rice et al., 2014), while amputation procedures—including rehabilitation and long-term care—range from $30,000 to $60,000 per patient (Kerr et al., 2019). Current standards of care involve wound debridement, dressings, glycemic control, and systemic antibiotics (Lipsky et al., 2020). However, these interventions are frequently insufficient. The predominant bacterial pathogens in DFUs include *S. aureus*, *Pseudomonas aeruginosa*, and *Escherichia coli* (Lipsky et al., 2020; Kale et al., 2023), with *S. aureus* being the most common etiological agent due to its capacity to survive both extracellularly and intracellularly (Liu et al., 2024). Gentamicin remains a widely prescribed empirical treatment (PLM, 2025; Amirrah et al., 2020); nevertheless, the increasing prevalence of antibiotic-resistant strains poses a major therapeutic challenge (Coşkun et al., 2024).

Conventional therapies face critical limitations: topical antibiotics often fail to penetrate infected tissues or reach intracellular reservoirs, while systemic administration is hindered by rapid clearance, frequent dosing requirements, and suboptimal tissue distribution (Chaves and Tadi, 2025a; NIH Office of Dietary Supplement, 2023a). These shortcomings compromise clinical efficacy and contribute to antimicrobial resistance. Consequently, there is an urgent need for innovative therapeutic approaches that combine antimicrobial potency with targeted drug delivery.

Nanoparticle-based systems, particularly exosomes, have emerged as promising candidates to overcome these challenges. Exosomes are nanoscale extracellular vesicles secreted by numerous cell types, including mesenchymal stem cells and complex biological sources such as bovine spleen extracts. In formulations like IMMUNEPOTENT CRP (ICRP), spleen-derived exosomes carry a diverse repertoire of bioactive molecules—including low-molecular-weight peptides, cytokines, and heat shock proteins—endowing them with potent immunomodulatory activity with affinity to leucocytes. Importantly, these vesicles preserve complex immune functions derived from their cellular origin, allowing modulation of the local microenvironment (García Coronado et al., 2024). Evidence indicates that exosomes accelerate wound healing by enhancing angiogenesis, regulating immune responses, and promoting tissue repair in chronic wounds, including DFUs (García Coronado et al., 2024; Zhang et al., 2018). These effects are mediated in part through intercellular communication and activation of key signaling pathways, notably AKT phosphorylation (García Coronado et al., 2024; Raghav et al., 2021).

Normal wound healing progresses through four coordinated phases: (a) hemostasis, involving platelet aggregation and fibrin clot formation; (b) inflammation, characterized by immune cell infiltration and cytokine release; (c) proliferation (2–10 days post-injury), marked by fibroblast activity, angiogenesis, and granulation tissue formation; and (d) remodeling, where type III collagen is gradually replaced by type I collagen, resulting in scar maturation, and increased tensile strength. In DFUs, this sequence is disrupted by chronic hyperglycemia, impaired angiogenesis, and persistent inflammation, leading to delayed or incomplete healing. Exosomes address these deficits by enhancing angiogenesis, promoting collagen deposition, and modulating immune activity (Raghav et al., 2021). Moreover, their intrinsic role as natural nanocarriers enables efficient delivery of therapeutic agents, improving stability, uptake, and bioavailability.

Exosome-based therapies hold potential to transform DFU management. Notably, the encapsulation of antibiotics within exosomes has been shown to increase antimicrobial efficacy against drug-resistant *S. aureus in vitro* (Xu et al., 2024; Yang et al., 2018). Given the high clinical and economic impact of infected diabetic ulcers, the development of such advanced strategies is of critical importance.

Accordingly, the present study aimed to evaluate the therapeutic potential of exosomes isolated from a bovine spleen leukocyte extract (IMMUNEPOTENT CRP), loaded with gentamicin, in reducing infection and accelerating wound repair in a diabetic mouse model.

## 2 Materials and methods

### 2.1 Exosome isolation from the IMMUNEPOTENT CRP and gentamicin encapsulation

IMMUNEPOTENT CRP exosomes were purified as previously mentioned (García Coronado et al., 2024) by centrifugation employing the kit ExoQuick (Invitrogen™ 4478359) and centrifuged at 10,000 × g for 1 h, at 4 °C, then resuspended in PBS 1X as indicated on the ExoQuick kit. In a previous study shape, size, and protein content including HSP90AA1, HSPA1A, HSPA2, TLN1, HMGB1, HSC70, and Vesicle-associated membrane protein-associated protein B among others were reported by our research team (García Coronado et al., 2023), confirming the expression of canonical exosomal markers. The ICRP is measured in units, and 1U is equivalent to 1,154.37 μg of protein (evaluated by BCA method). The concentration of exosomes was evaluated by BCA method (Thermofisher, Cat. No. A55860). For encapsulation, 25 μL of purified exosomes (105.7450 μg) were incubated with 1,269 μL (126,900 μg) of gentamicin (Pisa Agropecuaria®, Gentaerba® injectable, México,10g/100 mL; equivalent to 100 μg/μL) at 37 °C for 30 min. It was then mixed with 731 μL electroporation buffer containing 0.6 M sucrose (final gentamicin concentration after electroporation = 62.67 μg/μL), and subsequently electroporated in a disposable electroporation cuvette at 250 V and 125 μF using an electroporator (BioRad MicroPulser, United States). Exosomes were recovered for downstream analyses and applications. After electroporation process, the size and shape of exosomes were assessed by AFM (Supplementary Figure S1).

### 2.2 Evaluation of gentamicin encapsulation efficiency

After gentamicin encapsulation, exosomes were recovered by centrifugation (10,000 × g for 1 h, at 4 °C). Following resuspension in 1 mL of 1X PBS, the loaded exosome pellet was incubated for 45 min in 1 mL of 1X Triton lysis buffer (1:1 v/v) to achieve gentamicin release. The supernatant (containing the released gentamicin) and the pellet (resuspended in PBS) were placed in a new 96-well plate (in triplicate wells per time measurement) and analyzed at 280 nm.

We corrected the absorbance reading by subtracting the absorbance of a blank control. This blank contained exosomes that were not exposed to gentamicin but underwent the exact same processing steps: electroporation, resuspension in PBS, and lysis with Triton X-100. This ensured we only measured the gentamicin-specific absorbance, excluding any background absorbance from proteins or exosome particles themselves. The calibration curve was established with gentamicin and the percentage of encapsulation was calculated using the following (Hernández Martínez et al., 2019)

$$\text{Encapsulation }\left(\%\right)=\left(\left(\text{initial gentamicin}\right)\;–\;\left(\text{free gentamicin in supernatant}\right)/\left(\text{total gentamicin}\right)\right)\;X\;100$$

All measurements were performed in triplicate, and results are presented as mean ± standard deviation.

### 2.3 Gentamicin release under acidic and alkaline conditions

To determine gentamicin release, loaded exosomes (not treated with Triton X-100) were incubated in a 96-well plate with either pH 2 or pH 8 buffer (in triplicate, 1:1 per pH) at different time points (0, 10, 30, 60, and 90 min). After incubation, samples were centrifuged (10,000 × g for 10 min), and the absorbance of the supernatant was read at 280 nm. Blank corrections were applied using non-loaded exosomes incubated in the same pH buffers and processed identically. This ensures that absorbance due to EV proteins or scattering is removed from final values. The release percentage was calculated using the following Formula 19:

$$\text{Release }\left(\%\right)=\left(\text{free gentamicin at }a\text{ given time}\right)\;/\;\left(\text{encapsulated gentamicin}\right)\;X\;100$$

All measurements were performed in triplicate, and results are presented as mean ± standard deviation.

### 2.4 Diabetic mice model

The type 1 diabetic model was induced in six-week-old BALB/c female mice (26–30 g), which were kept at a constant temperature of 28 °C in a vivarium with *ad libitum* access to food. Diabetes was induced with the protocol of three (daily) doses of streptozotocin intraperitoneally (65 mg/kg of body weight dissolved in citrate buffer pH 4.5) after a 4-h starvation period. Two weeks after the last injection with STZ, a glucose tolerance curve was performed after a 12-h fast and a subsequent dose of oral dextrose (2 mg/kg of body weight). Blood glucose levels were measured every 30 min for 2 hours using an Accu-Chek Instant glucometer. Samples were obtained by puncturing the caudal vein. Mice with glucose levels >160 mg/dL were considered diabetic (García Coronado et al., 2024). All the experimental protocols were approved by the ethics and animal welfare committee of the College of Biological Sciences of the Autonomous University of Nuevo León (N° CEIBA-2018-013) and were conducted under veterinary supervision. The production, care, and use of experimental animals for all experiments adhered to the official Mexican standard NOM-062-ZOO-1999.

Mice were randomly assigned to experimental groups (n = 5 per group per day) in accordance with the approved study design and the maximum number of animals authorized by the Institutional Bioethics Committee. Mice were monitored longitudinally to evaluate wound closure following treatment with the formulation described below. Blood samples were collected from the same animals on days 0, 7, 14, and 21 to assess cytokine modulation (n = 5 per time point). Additionally, tissue samples were collected on days 7 and 14 for histological evaluation (n = 5 per time point).

#### 2.4.1 Bacterial inoculation and subsequent treatment of topical wounds

Mice were anaesthetized by intramuscular injection with ketamine (80 mg/kg) and xylazine (5 mg/kg). The backs of the mice were then depilated and disinfected with ethanol (70%). A dorsal wound was made by taking a piece of skin (0.5 × 0.5 cm).

To induce wound infection, an ATCC strain of *S. aureus* was reseeded on Petri dishes with blood agar for cultivation. Individual colonies were selected and grown in the nutrient broth. Using the McFarland scale, the concentration of *S. aureus* was adjusted to 10^7^ CFU by turbidity measurement. Finally, this suspension of 10^7^ CFU (100 µL) of *S. aureus* was inoculated directly onto the dorsal wound in mice on day 0. The bacterial suspension was left on the wound for 1 h before the corresponding treatments were administered. The negative (PBS) and positive control (Gentamicin) groups were infected with the same bacterial suspension (10^7^ CFU) under the same conditions.

On day 7 after wounding, the susceptibility of *S. aureus* to the different treatments was evaluated in a sterile environment. The wound was washed with sterile saline solution (100 µL), and a sample of the wash was then taken with a sterile swab. Collected samples were cultured on blood agar plates and incubated at 37 °C for 24 h to allow for bacterial growth. The growth of CFU was documented by photography on Petri dishes. A count was then performed using the “Colony Count” application to determine the exact number of CFUs on the plates (Promega, United States).

The treatments were applied as follow on the wound by adding drops gradually: PBS as negative control (5 doses total, one per day (100 µL)), Gentamicin as positive control (5 doses total, one per day (100 µL)), pellet of ICRP (5 doses total, one per day (100 µL)), ICRP (5 doses total, one per day (100 µL containing 1 U dissolved in injectable water)), exosomes of ICRP (5 doses total, one per day (100 µL containing the exosomes of 1U of ICRP)), and Exo-Genta (5 doses total, one per day (100 µL)).

### 2.5 Wound closure assessment

Histological evaluations were performed using five tissue measurements at different fields (n = 5). The excised tissue collected during wound creation served as the day 0 sample, while tissue harvested from the five mice per group sacrificed at day 21 was used for endpoint histological assessment. All animals received scheduled analgesia and were monitored daily for welfare; predefined humane endpoints were applied to ensure early euthanasia when necessary.

Animals underwent daily clinical observation for 21 days, during which wound closure was documented through serial photographic imaging. Additionally, wound dimensions were measured using a vernier caliper directly at the wound site in live mice following treatment. Wound closure was calculated using the following equation:

$$\text{Wound closure }\left(\%\right)=\left(A_{0}‐\;A_{t}\;\right)÷\left(A_{0}\;x\;100\right)$$

Where A_0_ is the wound area at time 0, and A_t_ is the wound area corresponding to each time point.

On day 0, all mice presented comparable physiological conditions and wound sizes. At this time point, the percentage of wound closure was set to 0% as a reference baseline. Each group consisted of 5 mice per time point (n = 5).

### 2.6 Assessment of collagen synthesis

Mice were anaesthetized as previously described and euthanized via cervical dislocation on days 7, 14, and 21 post-treatment. This method was chosen to avoid chemical overdose interference with the parameters analyzed. Dermal tissues from the wound site were excised, fixed in 10% formaldehyde for 24 h, and embedded in paraffin. Tissue sections (5 μm thick) were prepared and mounted on slides for subsequent deparaffinization and rehydration protocol. Collagen fibre expression was assessed using Masson’s trichrome staining. Micrographs were acquired at ×10 magnification using a Zeiss confocal microscope equipped with an Axiocam camera. Image analysis was conducted using ImageJ software with the FIJI plugin (version 1.54f; http://imagej.org) to quantify the percentage of expression per image field. The results were graphically represented using GraphPad Prism (v. 9.0.0). (GraphPad Software, San Diego, CA, United States). For each treatment group, three representative microscopic fields were photographed, and one representative image was selected for inclusion in the final figure.

### 2.7 Epithelial thickness, epithelial area, and cell number calculation

Skin tissue samples were collected from the scar site of treated mice on days 0, 7, 14, and 21 post-treatments. Hematoxylin and eosin (H&E) staining was performed to facilitate cell quantification by nuclear counting; all analyses were performed by counting the cells of the dermis section. Image analysis was conducted using the FIJI plugin of ImageJ software (v. 1.54f, http://imagej.org), employing the colour deconvolution function to isolate the purple hematoxylin signal. A binary mask image was generated using the watershed function to separate closely adjacent nuclei, which were then counted. Nuclei located at the edges of the field were excluded from quantification. Cell counts represent the mean values obtained from randomly selected fields per sample (n = 5), covering from the epidermis to the subcutaneous tissue, captured at ×40 magnification using a Zeiss confocal microscope equipped with an Axiocam camera. Data were visualized using GraphPad Prism (v. 9.0.0). (GraphPad Software, San Diego, CA, United States).

Images obtained from Masson’s trichrome staining for collagen expression analysis were further used to evaluate epithelial thickness and area, from the top to the bottom of the epidermis, characterized by a red-pink area at the edge of the tissue. Epithelial thickness was measured using FIJI software by calibrating the image scale according to the magnification (2000 pixels = 1 μm). For each sample image (n = 5), five random linear measurements were taken across the epithelium using the “Straight” tool. Measurements were acquired using the “Measurement” command under the “Analyse” tab, and the results were processed in GraphPad Prism (v. 9.0.0). (GraphPad Software, San Diego, CA, United States).

To calculate epithelial area, the images were similarly scaled and analyzed. The “Polygon selections” tool was used to delineate the entire epithelium in each sample, and area measurements were extracted via the “Measurement” command. Subsequent data analysis was performed using GraphPad Prism (v. 9.0.0). (GraphPad Software, San Diego, CA, United States).

Granulation tissue thickness was also assessed by identifying the tissue, drawing three random lines (2000 pixels = 1 μm) across its thickness per sample using the “Straight” tool in FIJI, and obtaining measurements with the same procedure, this tissue is characterized by a darker blue colour accumulation and infiltration of immune cells. Results were analyzed in GraphPad Prism (v. 9.0.0). (GraphPad Software, San Diego, CA, United States) (n = 5).

Wound healing was evaluated using a scoring system adapted from Greenhalgh et al. (1990), as shown in Table 1.

**TABLE 1:** Scoring of histology sections for the skin tissue from wound site stained with Trichrome staining (Greengalgh, 1990).

Score	Criteria
1–3	None to minimal number of cells. No granulation tissue present, nor epithelial travel
4–6	Thin/immature granulation tissue dominated by inflammatory cells. Few fibroblasts, capillaries or collagen deposition. Minimal epithelial migration
7–9	Moderately/thick granulation tissue. Dominated by inflammatory cells to fibroblast and collagen deposition. Extensive neovascularization. Epithelium from minimal to moderate
10–12	Thick vascular granulation tissue, dominated by fibroblasts and extensive collagen deposition. Epithelium tissue partially/completely covering the wound

### 2.8 Sera pro-inflammatory cytokines

Blood samples were collected via cardiac puncture from mice on days 7, 14, and 21 post-treatments. Serum was collected to quantify pro-inflammatory cytokine levels using the BD Cytometric Bead Array (CBA) Mouse Inflammation Kit (Catalog No. 552364), following the manufacturer’s instructions. Data were analyzed using FlowJo software, applying the four-parameter logistic (4PL) curve fitting method for extrapolation. Expression values below the limit of detection (<LOD in Figure 7) were still included in the statistical analysis, as they were consistent and relevant to the hypothesis. The limits of detection (LOD) for the cytokines were as follows: IL-6 (5 pg/mL), IL-10 (17.5 pg/mL), MCP-1 (52.7 pg/mL), IFN-γ (2.5 pg/mL), TNF (7.3 pg/mL), and IL-12p70 (10.7 pg/mL). Data were subsequently visualized using GraphPad Prism (v. 9.0.0). (GraphPad Software, San Diego, CA, United States). All experiments were performed in triplicate (n = 5).

### 2.9 PI3K-AKT pathway activation by Exo-Genta

Samples obtained on day 7 were analysed to evaluate the phosphorylation status of key components in the PI3K-AKT signaling pathway using the VECTASTAIN Elite ABC HRP kit (Vector Laboratories, PK-6100) according to the manufacturer’s guidelines. The primary antibodies employed included AKT-P (PA5-39725), FOXO-P (PA5-118528), P21-P (PA5-99373), and TSC2-P (PA5-104916) (Thermo Fisher), and as a sign of activation of the pathway the downstream expression of type I collagen was assessed (Santa Cruz, sc-59772). Marker expression was visualized by the brown precipitate resulting from the enzymatic reaction of the DAB substrate (DAB Substrate Kit, Peroxidase [HRP], Vector Laboratories). Slides were photographed at ×40 magnification using a Zeiss confocal microscope equipped with an Axiocam camera and processed in ImageJ software with the FIJI plugin (v. 1.54f, http://imagej.org). To separate and measure the brown coloration caused by DAB, the color deconvolution function was used. Background noise was then removed with the auto-treshold function. The percentage of DAB expression (brown colouration) was subsequently analysed. To quantify the percentage expression of each marker, five randomly selected images were obtained from distinct fields per treatment slide, encompassing the epidermis, dermis, adipose tissue, and, when possible, superficial muscle tissue. We selected one representative image per group for figure assembly and graphed expression data using GraphPad Prism (v. 9.0.0). (GraphPad Software, San Diego, CA, United States).

### 2.10 Statistical analysis

Statistical analyses were performed using GraphPad Prism (v. 9.0.0). (GraphPad Software, San Diego, CA, United States)., Prior the comparison against control and between groups a normality distribution test was performed (Shapiro Wilk, Kolmogorov Smirnov, or D’Agostino & Pearson tests p < 0.05). Statistical significance was determined using a two-way ANOVA test, followed by a Tukey multiple comparison test *post hoc* or T and Wilcoxon test (as applicable), with a significant difference denoted by p < 0.05 and data are expressed as the mean ± standard deviation.

## 3 Results

### 3.1 Exosomes as a delivery vehicle

**FIGURE 1:**
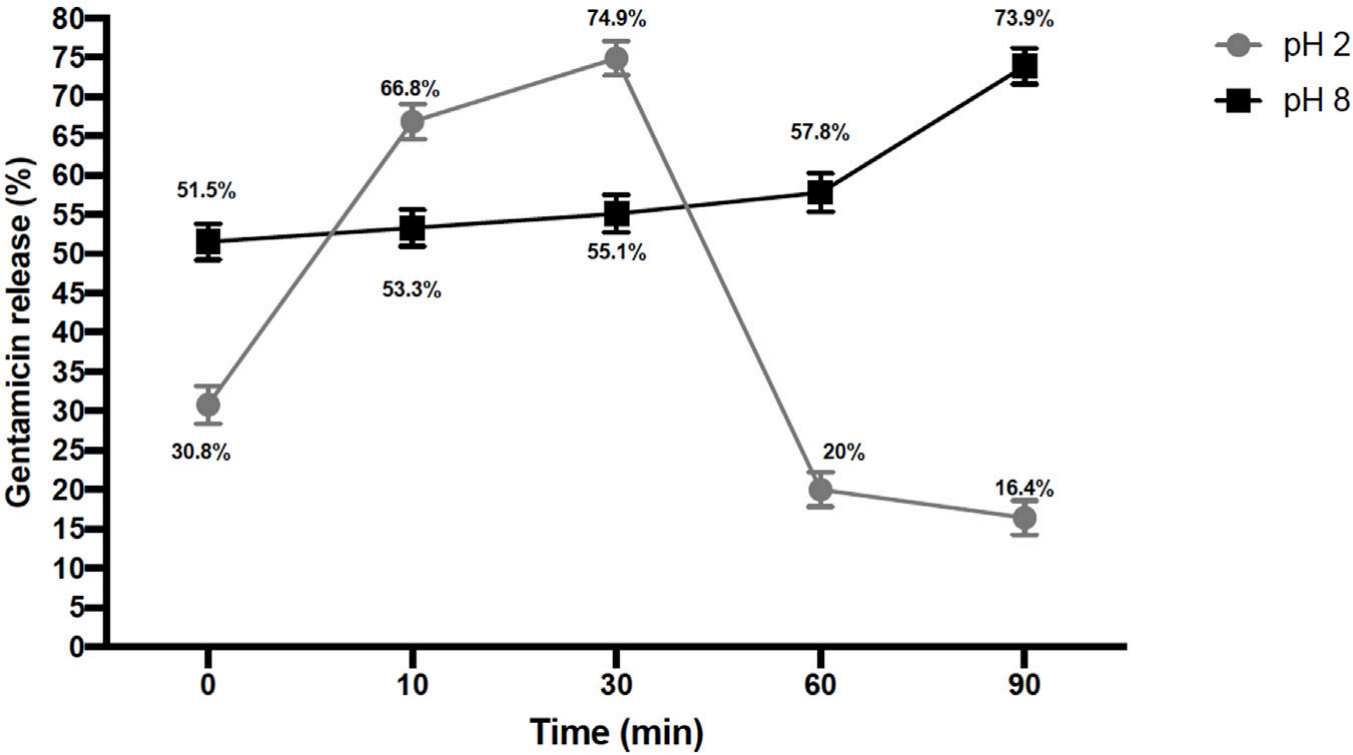
Gentamicin release curve. Gentamicin absorbance at time 0, 10, 30, 60, and 90 min in buffers with pH2 and pH8, mean values ±SD.

Gentamicin release kinetics from exosomes were evaluated at pH 2 and pH 8 (Figure 1). At pH 2, a rapid increase in gentamicin release was observed between 10 and 30 min, reaching a peak release of 74.9% at 30 min, compared to 16.4% at time zero. In contrast, at pH 8, gentamicin exhibited a sustained and controlled release profile, characterized by an initial release of 51.5% at time zero, which progressively increased to a peak release of 73.9% at 90 min. These results indicate that gentamicin release from exosomes is efficient under both conditions; however, release at basic pH appears to enhance the drug’s half-life more effectively. The encapsulation efficiency of gentamicin in exosomes, calculated using the equation y = −7.03716x + 673.95381 derived from absorbance values, was determined to be 31.85%.

### 3.2 The treatments of ICRP and its derivatives accelerate wound closure

**FIGURE 2:**
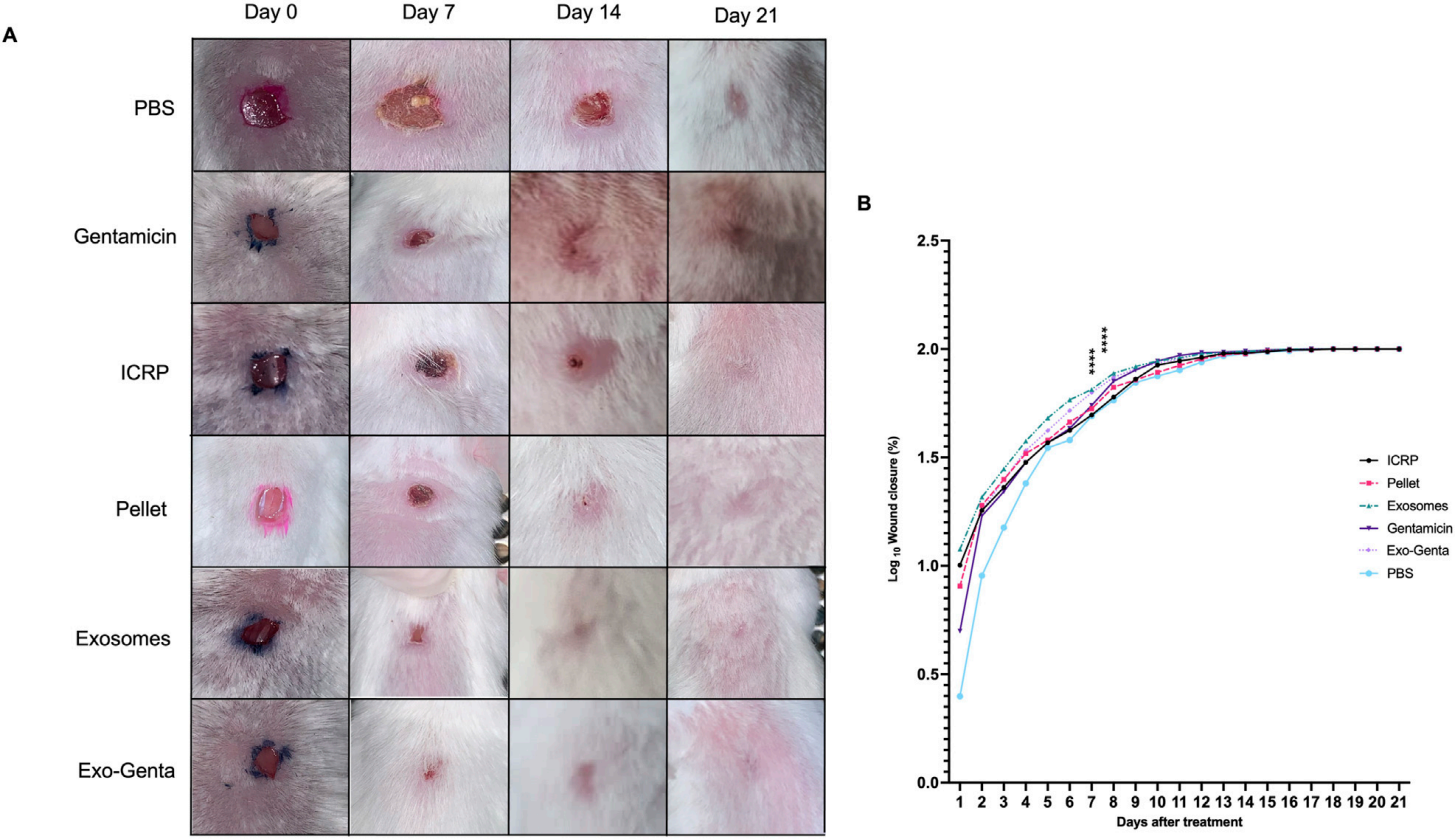
The exosome and Exo-Genta-treated groups showed accelerated wound closure. **(A)** Representative photographs (taken at days 0, 7, 14, and 21) of dorsal wounds in female BALB/c diabetic mice. **(B)** The wound area was assessed daily for 21 days. Log base 10. 2-way ANOVA, Tukey’s statistical test was performed ****p < 0.0001, mean values ±SD.

The formation of a scab on the wound was observed in the initial days post-treatment. By day 7 post-mortem, the excised wound area revealed the presence of pus beneath the dermis. Topical treatments facilitated dorsal wound healing, with exosome, gentamicin, and exosome–gentamicin formulations exhibiting the highest healing rates during the initial 12 days (Figures 2A,B) (p < 0.0001). In contrast, healing percentages were progressively lower in the ICRP, pellet, and PBS treatment groups (Figures 2A,B).

Exosome-treated wounds exhibited the highest closure rates until day 9 (p < 0.0001). From day 10 onward, healing rates among treatments converged, with the constant being that the PBS-treated wounds showed the least closure (Figures 2A,B). By day 17, complete wound closure was achieved in the exosome, gentamicin, and exosome-gentamicin groups. In contrast, other treatments reached full closure subsequently. Tukey’s test revealed significant differences (p < 0.0001), notably between the PBS group and the exosome, gentamicin, and exosome-gentamicin groups, as well as between the ICRP and pellet groups.

Furthermore, exosome and exosome-gentamicin treatments facilitated faster hair regrowth at the wound site, as observed by clinical examination, thereby enhancing scar regeneration. Furthermore, 100% wound closure was completed on day 17 in the exosome, exosome-gentamicin, and gentamicin treatments, while the remaining treatments closed completely from day 18 onwards.

### 3.3 The Exo-Genta treatment reduces the bacteria in the wound site

**FIGURE 3:**
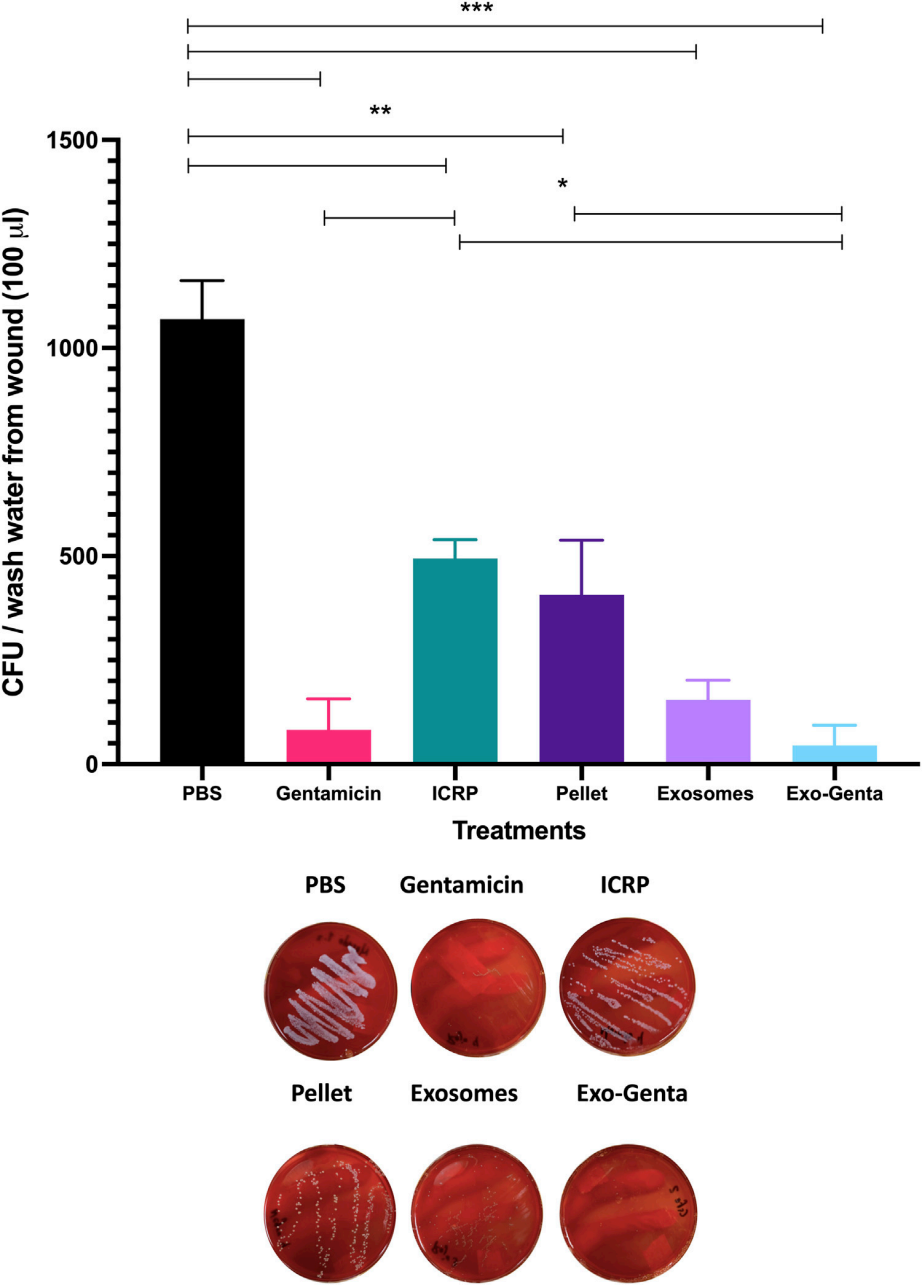
The Exo-Genta treatment promoted the decrease of *S. aureus* CFU in topical wounds of female BALB/c diabetic mice. Samples were taken from the dorsal wound on day 7 with saline solution to be inoculated onto blood agar plates and incubated for 24 h. Tukey test (p < 0.05). *p < 0.05, **p < 0.01, ***p < 0.001, n = 5 female BALB/c mice per group. Mean values ±SD.

The control group (PBS) exhibited the highest bacterial load, with an average colony-forming unit (CFU) count of 1,070. ICRP treatment decreased the amount at the wound site (497 CFU, p < 0.01), but not as well as the positive control treatment with a significant difference (gentamicin) (p < 0.001) (Figure 3). The exo–genta treatment group showed the lowest CFU count, averaging 45 CFU, with a highly significant difference (p < 0.001). A marked reduction in bacterial load was observed across the remaining treatment groups: exosomes (154 CFU), gentamicin (83 CFU), and exo–genta (45 CFU), with all differences showing high statistical significance compared to negative control (p < 0.001) (Figure 3). A significant difference was observed when comparing the ICRP and exo–genta treatment groups, suggesting that the enhanced antimicrobial activity may be attributed to the encapsulation of gentamicin (p < 0.05) (Figure 3). Nevertheless, no significant difference was observed between exosomes and exo-genta treatment (p < 0.05)

### 3.4 The exosomes, Exo-Genta isolated from the ICRP induce the expression of collagen in the wound site

**FIGURE 4:**
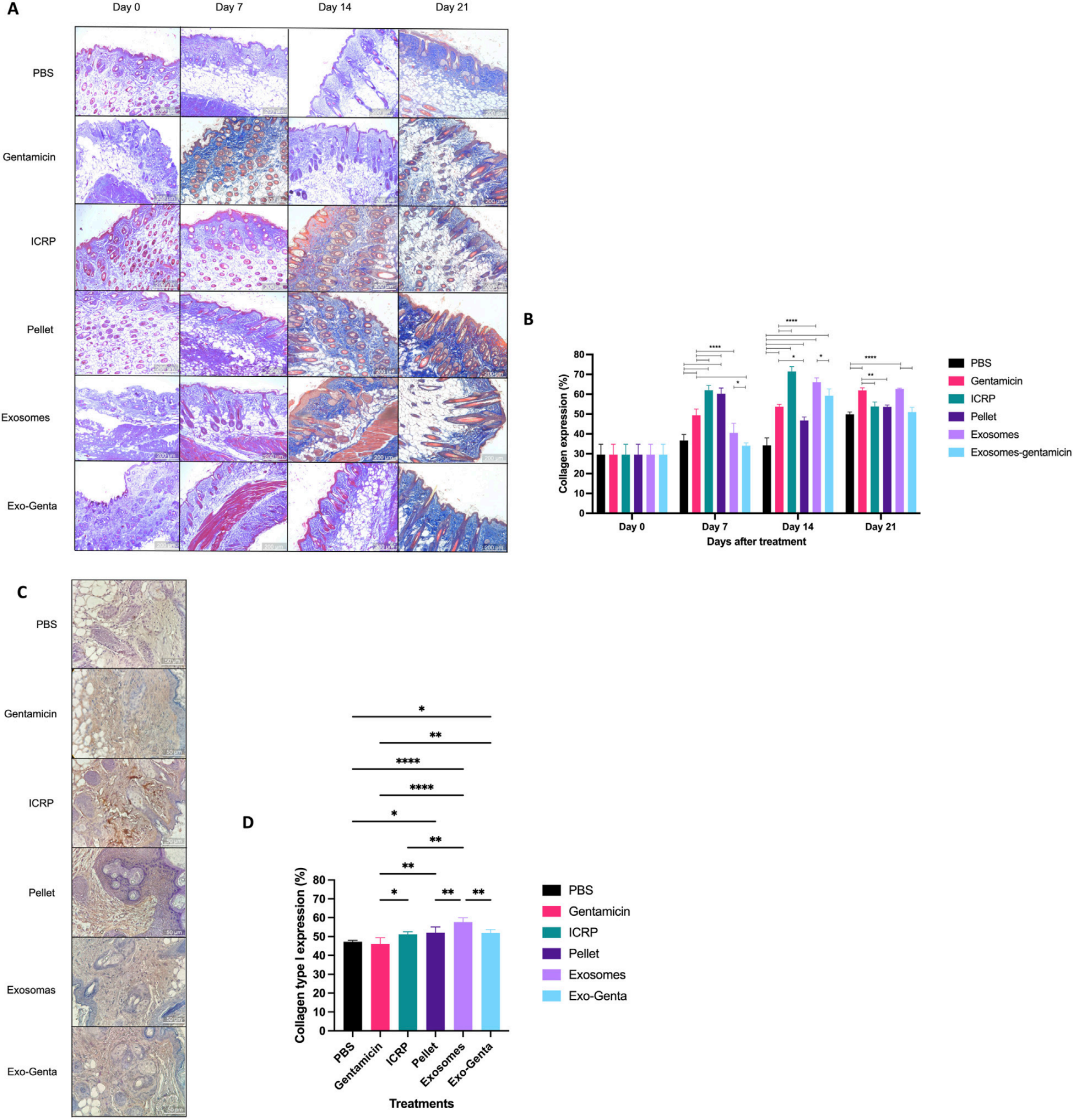
Masson trichrome stain of skin tissue from the wound area and collagen type 1 expressions by IH. **(A)** Tissues correspond to days 0, 7, 14 and 21 of the different groups. Blue color indicates the presence of collagen fibers, red color indicates muscle fibers, erythrocytes, and keratinocytes (10x). **(B)** The images were analyzed using the ImageJ software with the FIJI plugin color deconvolution function to determine the percentage expression of collagen fibers, with the mean values displayed in the graph. Statistical analysis was conducted using Tukey’s test (n = 5) the letters establish statistic difference against negative control (p < 0.05). Mean values ±SD. **(C)** Representative 10X images (amplified in the figure) were captured using a Zeiss microscope of IH against type I collagen. **(D)** Marker expression was quantified with ImageJ, and mean values were plotted. Statistical significance was assessed using Tukey’s test (n = 5): *p < 0.05, ****p < 0.0001. Mean values ±SD. *p < 0.05, **p < 0.01, ***p < 0.001, ****p < 0.0001.

A Masson trichrome stain was performed to evaluate the production of collagen fibres in the wound area after treatment (Figure 4). On day 7, the ICRP and its parts, as pellets, exosomes, and Exo-Genta treatments, showed greater organization of the dermis and subcutaneous tissue compared to the controls (p < 0.05) (PBS and Gentamicin). In the control treatment could be clinically observed that the tissues were disorganized from the epidermis to the subcutaneous layer; compared with the exosomes and exosome-gentamicin treatment where the layers were observed more organized (Figure 4). The ICRP, exosome, and exosome-gentamicin treatments showed a greater amount of collagen fibres, hair follicles, and blood vessel formation in greater numbers (p < 0.05), which are indicators of good healing (Figures 2, 4). At day 14, all the treatments increasing the expression of collagen fibres including the exosomes and Exo-Genta treatments. On the other side, at day 21, the exosomes treatment showed the highest collagen production compared to the rest of the treatments, and the day showed a higher regeneration of the wound and tensile properties due to collagen fibres presence (p < 0.05) (Figure 4B). But the treatment with the best collagen production was the group treated with ICRP (p < 0.05) (Figure 4).

By day 21, all the treatments started decreasing the amount of collagen fibres at the wound site statistically equal to negative control, except for exosomes treatment (p < 0.05), otherwise, all the ICRP, its parts and itself increased the collagen production at earlier days (day 7 and 14) than the negative control indicating the advance of the first phases of the wound healing process (granulation) (p < 0.05) (Figure 4). Another indicator of wound healing is the presence of keratin fibres (red fibres), as observed in Figure 4B. The specific production of type 1 collagen is analyzed in day 7 and it could be observed that the treatment with most type 1 collagen expression was the exosome treatment (Figure 4D) at earlier days than the rest of the treatments, accelerating the wound healing phases.

### 3.5 The ICRP and its parts accelerate wound healing through the phosphorylation of AKT

**FIGURE 5:**
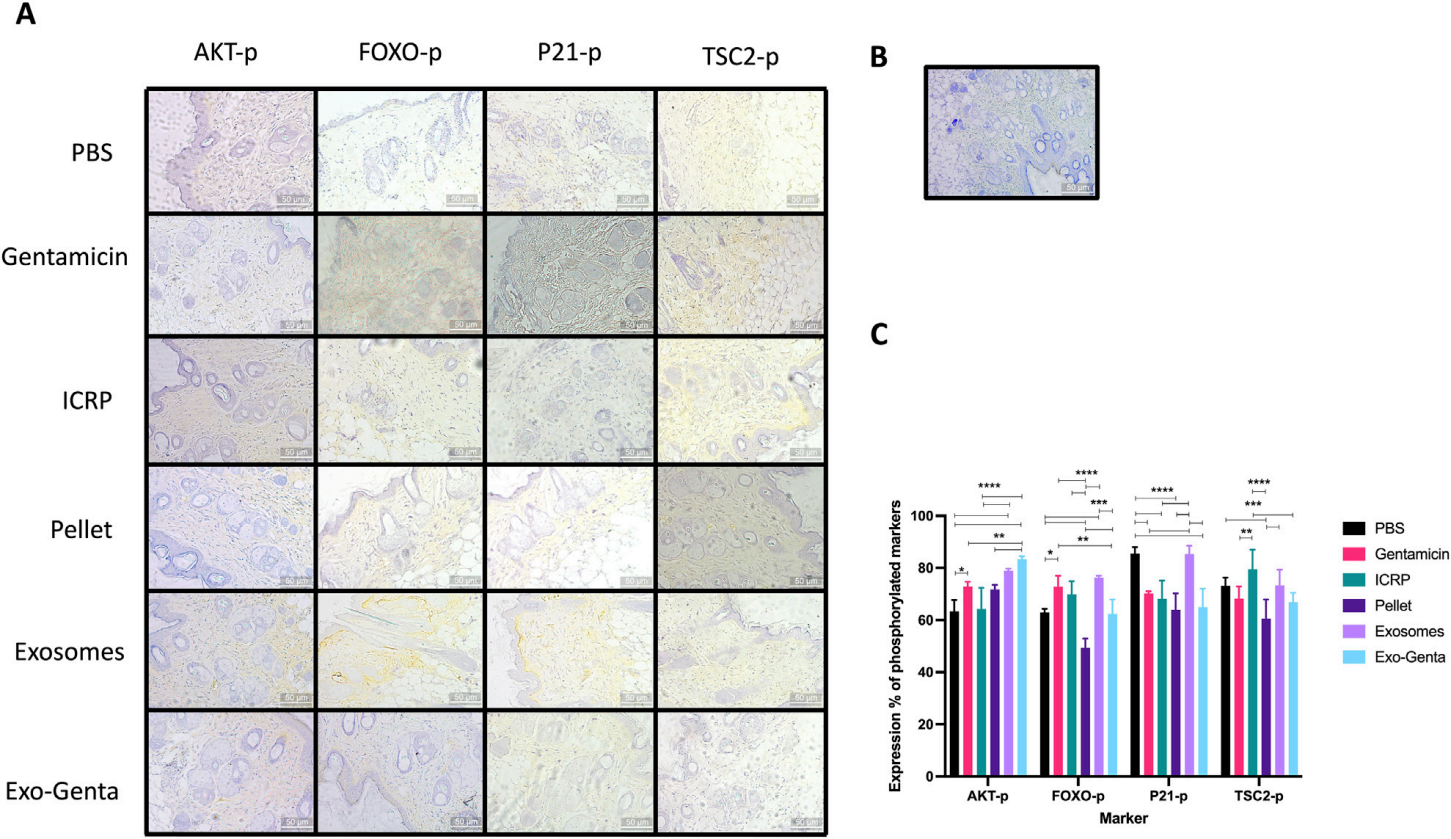
ICRP, pellet, exosomes, and Exo-Genta increased AKT phosphorylation. Wounds were treated topically for 5 days, and on day 7, skin samples were collected, fixed in 10% formaldehyde, and analyzed by IH with hematoxylin counterstaining. **(A)** Representative 10X images (amplified in the figure) were captured using a Zeiss microscope. **(B)** Representative photo of IH in tissue without primary antibody (10X Zeiss microscope. **(C)** Marker expression was quantified with ImageJ, and mean values were plotted. Statistical significance was assessed using Tukey’s test (n = 5): *p < 0.05, **p < 0.01, ***p < 0.001, ****p < 0.0001. Mean values ±SD.

Diabetic mice with infected wounds treated with ICRP components exhibited increased phosphorylation of the AKT marker, associated with cellular survival, proliferation, and growth pathways, showing a significant difference compared to the negative control (p < 0.05) (Figure 5). The positive control group treated with gentamicin exhibited a 72.82% AKT-phosphorylated expression with statistical difference against negative control (p < 0.05) (Figure 5B); The mice treated with exosomes and Exo-Genta expressed more AKT-p than gentamicin treatment (p < 0.05) (Figure 5B). The treatment that significantly induced the highest activation of AKT compared to the negative control were exosomes (78.92%) and Exo-Genta (83.4%) (p < 0.0001) (Figure 5B).

An important downstream target of AKT phosphorylation is the transcription factor FOXO, which also undergoes phosphorylation as part of the signaling cascade. The treatments that exhibited higher expression of FOXO phosphorylation were positive control (gentamicin) (72.75%) and exosomes (76.24%), both with a significant difference compared to negative control (p < 0.0001) (Figure 5B).

When P21 and TSC2 are phosphorylated, the negative regulation of the AKT pathway is activated, inhibiting the cell cycle. As observed in Figure 2B, the ICRP and its parts accelerated the wound healing process. As shown in Figure 5B, all treatments and the positive control demonstrated reduced expression of phosphorylated P21 (p < 0.01), except for the exosome treatment group, statistically the same as the negative control. The pellet treatment exhibited the lowest expression of phosphorylated p21 (63.97%), showing a significant reduction compared to the negative control (85.46%, p < 0.0001) (Figure 5B). The other negative marker of pathway activation is TSC2 phosphorylation; it could be observed a significant difference when comparing pellet (61.83%) with negative control (72.5%) (p < 0.05). When compared against positive control (68.26%), and ICRP (79.47%), p < 0.01), increased the expression of TSC2-p (Figure 5B).

### 3.6 The ICRP and its parts increase the regeneration of diabetic-infected wounds

**FIGURE 6:**
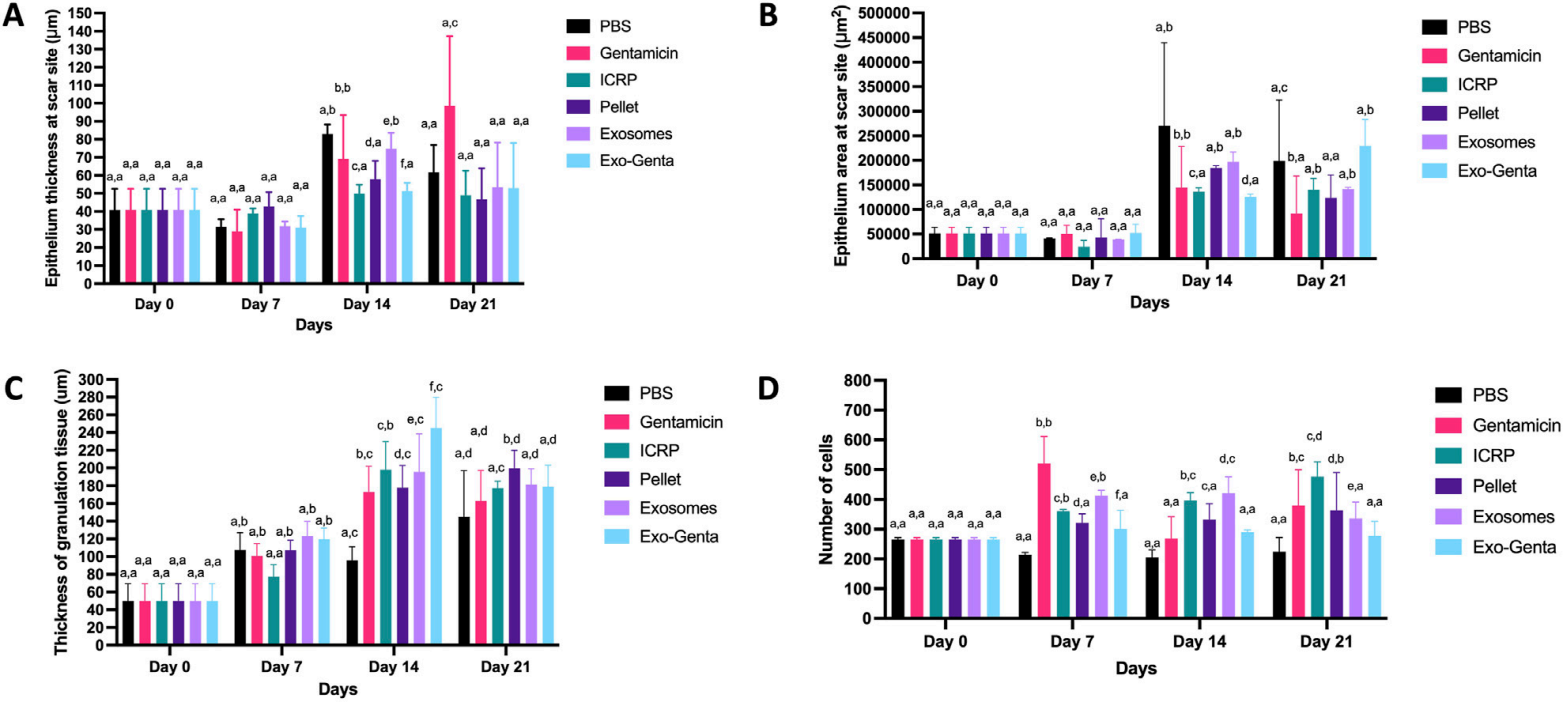
The ICRP, pellet, exosomes, and Exo-Genta increase wound regeneration. The wound area was assessed for **(A)** Epithelium thickness at the scar site (μm, in 40X representative photographs, 2000 pixels = 1 µm). **(B)** Epithelium area at the scar site (μm^2^, in 40X representative photographs, 2000 pixels = 1 µm). **(C)** Thickness of granulation tissue at the scar site (μm, in 40X representative photographs, 2000 pixels = 1µm). **(D)** Number of cells at the scar site in the dermis zone (in 40X representative photographs). Statistical analysis was conducted using Tukey’s test (n = 5) the letters establish statistic differences within and between groups (p < 0.05). Mean values ±SD.

The epithelium area indicates the progression of wound healing. On day 7, epithelial thickness was reduced; however, statistical analysis revealed no significant difference compared to day 0 (p < 0.05). Regarding tissue thickness, the PBS-treated group exhibited the highest value on day 14 (p < 0.05) (Figure 6A). By day 14, all treatment groups showed increased epithelial thickness, with statistically significant differences compared to the negative control at day 0, as well as among the treatment groups themselves (p < 0.05) (Figure 6A). Among the treatments, exosomes resulted in the greatest epithelial thickness (77.41 μm), second only to the PBS group (99.23 μm) (Figure 6A) (p < 0.05). The epithelial area was reduced on day 7; however, by days 14 and 21, all treatment groups showed an increased epithelial area compared to day 0 and 7 (p < 0.05). Significant differences were observed for gentamicin (144,200 μm^2^ on day 14), exosomes (197,039 μm^2^ on day 14), and pellet (184,506 μm^2^ on day 21) treatments, relative to the baseline value on day 0 (51,363.2 μm^2^) (p < 0.05) (Figure 6B) the differences in the techniques can be observed at the Supplementary Figure S2.

Granulation tissue formation is indicative of progression into the proliferative phase of wound healing; increased thickness reflects more advanced tissue regeneration. As shown in Figure 6C, no significant differences in granulation tissue thickness were observed within treatments on days 0 and 7 (p < 0.05). However, by day 14, all treatments demonstrated significant increases, with ICRP (198.02 μm), exosomes (195.6μm), and Exo-Genta (245.05 μm) showing the greatest differences compared to the negative control (95.73 μm) (p < 0.05). By day 21, significant changes were observed within each treatment group across time points. On day 21, the pellet-treated group exhibited the highest granulation tissue thickness (199.52 μm) (p < 0.05). Overall, the exosome–gentamicin treatment on day 14 showed the greatest granulation tissue thickness observed throughout the study (245.05 μm) (p < 0.05).

Cell counts in the dermal region were analyzed as an indicator of wound regeneration. By day 7, the gentamicin-treated group (positive control) exhibited the highest number of cells at the wound site (641.3 cells), with a statistically significant difference (p < 0.05) (Figure 6D). When the monitoring ended, all treated mice exhibited a higher number of cells in the dermis compared to day 0 (p < 0.05) (Figure 6D). At day 7, all treatments showed a greater number of cells with a significant difference compared to negative control PBS (p < 0.05) (Figure 6D). Among the treatment groups, ICRP demonstrated a progressive increase in cell number from day 0, reaching the highest count by day 21 (453.5 cells), with a significant difference observed (p < 0.05) (Figure 6D). According to Greenhalgh et al. (1990) (Greenhalgh et al., 1990), ICRP and its components may accelerate wound regeneration by promoting early collagen deposition, as reflected in Table 2.

**TABLE 2:** Skin tissue scoring for wound regeneration.

Treatment	Score day 7	Score day 14	Score day 21
PBS	3	5	7
Gentamicin	4	8	8
ICRP	5	8	9
Pellet	5	6	7
Exosomes	7	9	10
Exo-genta	7	9	10

### 3.7 The ICRP and its parts modulate the inflammation in diabetic mice with infected wounds

**FIGURE 7:**
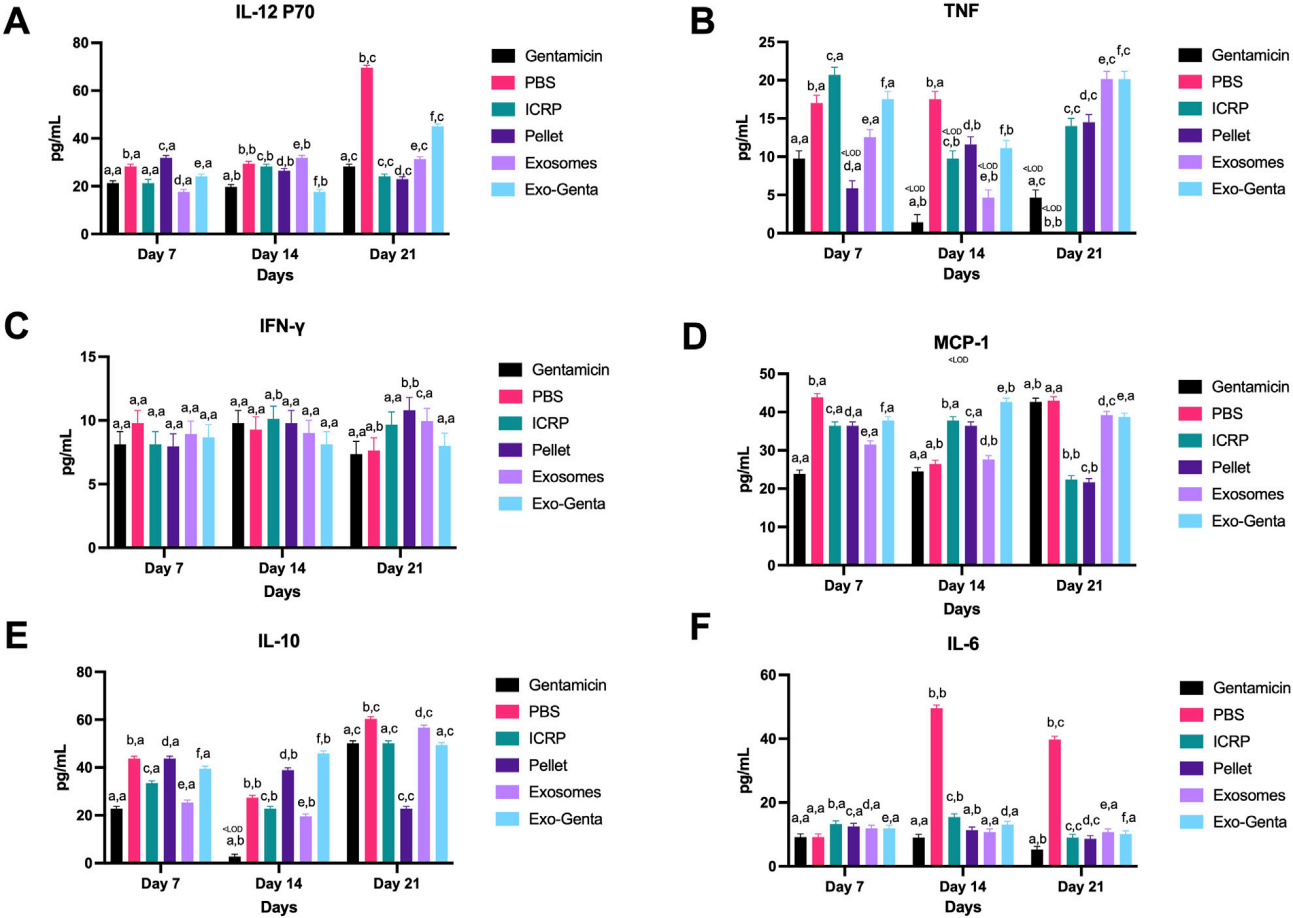
Inflammatory cytokine expression modulation in diabetic mice treated topically at the wound site. Blood was obtained on days 7, 14, and 21 and sera separated and evaluated through flow cytometry. **(A)** IL-12 p70. **(B)** TNF. **(C)** IFN-γ. **(D)** MCP-1. **(E)** IL-10. **(F)** IL-6. Tukey test (*p* < 0.05) (n = 5). The letters establish statistic differences within and between groups, <LOD: below Limit Of Detection point. Mean values ±SD.

Diabetic patients are characterized by a chronic inflammatory microenvironment that affects the progression of wound healing phases. Based on these findings, the inflammatory status was evaluated in mice treated with ICRP or its individual components. On day 7, the treatment that exhibited a lower expression of IL-12P70 (pg/mL) was exosomes (17.61 pg/mL) with a significant difference compared to the positive control (p < 0.05) (Figure 7A). On day 14, the treatment that diminished the expression was Exo-Genta (17.61 pg/mL), with a significant difference compared to the positive control (p < 0.05) (Figure 7A). On day 21, IL-12p70 expression, showed an increase in PBS and Exo-Genta groups compared to the positive control, with significant differences observed (p < 0.05) (Figure 7A).

The TNF-α is considered pro-inflammatory, and at the beginning of the treatment on day 7, the group that exhibited a decreased expression of TNF-α was pellet (5.86 pg/mL), with a significant difference against positive control (9.76 pg/mL) (p < 0.05) (Figure 7B), opposite to the rest of the treated mice on the other groups that showed an increased in the expression (Figure 7B). On day 14, the groups that showed the lower expression were positive control (1.42 pg/mL) and exosomes (4.66 pg/mL), and the group with the highest expression was PBS (17.54 pg/mL) with a significant difference compared to the positive control (p < 0.05) (Figure 7B). By day 21, TNF-α expression varied depending on the extent of component isolation, with significant differences observed (p < 0.05). Notably, the PBS group showed an absence of TNF-α expression, which was significantly different compared to the positive control (p < 0.05) (Figure 7B). Through the progression of days, the treatment exhibited a decreased expression, but after the treatment doses were done, increased the expression, with a significant difference between the pellet and Exo-Genta (p < 0.05) (Figure 7B)

IFN-γ serum concentrations did not vary over the days. On day 21, pellet (10.8 pg/mL) and exosome (9.95 pg/mL) treatments demonstrated a significant increase in IFN-γ expression compared to controls (p < 0.05) (Figure 7C). Mice treated with pellet exhibited a significant increase in IFN-γ expression over time (p < 0.05) (Figure 7C). The higher MCP-1 expression was observed in the negative control (p < 0.05) (Figure 7D). By day 21, the ICRP (22.41 pg/mL) and pellet (21.64 pg/mL) decreased the concentration of MCP-1 compared to positive control (gentamicin) (p < 0.05), but the ICRP-part exosomes (39.18 pg/mL) and Exo-Genta (38.73 pg/mL) increased compared to ICRP and pellet, nevertheless, lower than controls (p < 0.05). On day 14, ICRP and its components exhibited sustained elevated MCP-1 expression levels (27.65–42.63 pg/mL) compared to controls (24.52–26.45 pg/mL), with significant differences observed (p < 0.05) (Figure 7D). Among the treatments, Exo-Genta maintained consistently high MCP-1 expression relative to controls throughout the study period (p < 0.05) (Figure 7D).

IL-10 expression was significantly modulated (Figure 7E) (p < 0.05). On day 7, all treated mice exhibited significantly increased expression compared to the positive control (p < 0.05) (Figure 7E), with PBS and pellet treatments showing the highest levels (43.78 pg/mL each) (p < 0.05) on day 7 (Figure 7E). On day 14, a similar expression to day 7 was observed but with decreased values (Figure 7E), outstanding the increase in expression of Exo-Genta and pellet treatments compared to positive control (p < 0.05). By day 21, the pellet treatment showed a lower expression (22.79 pg/mL) with respect to negative and positive controls (p < 0.05). The treatments with significant difference compared to the negative control, with increased expression, were PBS (60.32 pg/mL) and exosomes (56.67 pg/mL) (p < 0.05) (Figure 7E). The treatment that continuously significantly increased (p < 0.05) the concentration of serum IL-10 over days was Exo-Genta (39.59 pg/mL to 49.46 pg/mL) (Figure 7E).

The pro-inflammatory IL-6 cytokine was diminished in treated mice except in the PBS group (49.58 pg/mL on day 14). On day 7, the treated groups exhibited a slight but significant increase in expression compared to the control (p < 0.05) (Figure 7F). On day 14, all treatment increase the expression compared to the positive control except for the groups treated with pellet and exosomes.

## 4 Discussion

Infection in chronic diabetic wounds is a major complication that increases the risk of delayed healing and limb amputation. In this study, we demonstrated that gentamicin encapsulated in exosomes derived from IMMUNEPOTENT CRP (ICRP), is employed as nanocarriers, reduced *S. aureus* infection and promoted wound healing in diabetic mice. This effect may be attributed to the ability of exosomes to deliver antibiotics into infected tissues and potentially into intracellular reservoirs of *S. aureus*, a pathogen known to persist both extracellularly and intracellularly (Cordeiro et al., 2000; Bravo-Santano et al., 2018; Guo et al., 2024). Previous studies have reported that encapsulation of therapeutic agents—such as gentamicin or insulin—within nanoparticles or exosomes enhances their bioavailability and regenerative effects (García Coronado et al., 2023; Cordeiro et al., 2000; Guo et al., 2024), which aligns with our findings. The release profile showed that 74.9% of gentamicin was released at pH 2 after 30 min (Figure 1), followed by a decrease at 60 minutes—likely due to acid-mediated degradation (Chaves and Tadi, 2025b). Despite this, the incomplete release suggests that exosomal encapsulation conferred partial protection to the drug, potentially improving its stability and half-life under acidic conditions characteristic of infected wounds. Gentamicin has a systemic half-life of approximately 30–90 min after intravenous administration and is typically cleared within 24 h (Chaves and Tadi, 2025b; Siber et al., 1975). While intravenous delivery offers prolonged bioavailability, it is invasive and impractical for outpatient care. Topical administration, although more accessible, struggles with limited tissue penetration and inconsistent absorption, especially in deep or chronic infections (NIH Office of Dietary Supplement, 2023b). These limitations underscore the need for local delivery systems capable of maintaining therapeutic antibiotic concentrations at the infection site. Notably, the acidic microenvironment of infected wounds triggered rapid release, with 74.9% of gentamicin released at pH 2 within 30 min (Figure 1), suggesting improved local drug retention and an extended therapeutic window with reduced dosing frequency (Abbasi et al., 2023). This improvement is likely associated with effective infection control during the early phase of treatment, especially in groups receiving ICRP-derived exosomes. Early pathogen clearance is critical for proper wound healing, as persistent infection impairs tissue regeneration (Nagle et al., 2003).

Notably, groups treated with exosomes, gentamicin, or the combined formulation demonstrated markedly reduced CFU counts of *S. aureus* on blood agar, with bacterial loads up to five times lower than PBS-treated controls. This antimicrobial effect is consistent with prior studies showing that exosomes can deliver antibiotics like vancomycin or lysostaphin into infected macrophages, reducing both intracellular bacterial load and host cytotoxicity (Yang et al., 2021). Moreover, exosomes derived from immune system cells possess the ability to exert biological functions similar to those of their cells of origin. For instance, macrophage-derived exosomes have been shown to control intracellular infections caused by *S. aureus* (Guo et al., 2024; Yang et al., 2021). It is important to note that ICRP is isolated from a leukocyte extract, and thus its exosomes are expected to retain biological activities comparable to those of their parental cells. In addition, the ICRP has been reported to exhibit bacteriostatic activity (Franco-Molina et al., 2006), our findings suggest that exosomes derived from ICRP not only retain but enhance this effect through gentamicin encapsulation, supporting their dual role as both antimicrobial agents and delivery vehicles. Further supporting the antimicrobial efficacy of exosome-based therapies, garlic-derived exosomes have been shown to reduce *S. aureus* CFU counts and accelerate wound healing in porcine models infected with multidrug-resistant strains (Zhou et al., 2024). Similarly, milk-derived exosomes encapsulating isobavachalcone or polymyxin have demonstrated enhanced biocompatibility and drug solubility while preserving antimicrobial potency (Xu et al., 2024). In line with these findings, gentamicin-loaded exosomes derived from ICRP exhibited the lowest CFU counts in infected wounds, confirming their superior therapeutic performance. *S. aureus* besides infecting epithelial cells, can also infect immune system cells, such as macrophages (Touaitia et al., 2025); it must be remembered that the exosomes preserve biological functions of the cell of origin, given that the exosomes from the ICRP come from a leucocyte extract, probably conserve the ability of interacting with macrophages entering to these cells and modulate healing and delivering intracellularly gentamicin.

Beyond infection control, IMMUNEPOTENT CRP has been shown to modulate cytokine expression and reduce inflammation in clinical settings such as third molar extractions (Franco-Molina et al., 2016). Such action—controlling inflammation—may prevent disruption of key metabolic pathways involved in tissue regeneration. This hypothesis is supported by the increased expression of collagen and keratin during early healing stages in treated groups (Su et al., 2024; Hobbs et al., 2012; Konop et al., 2029). Notably, by day 14, the Exo-Genta group showed the highest collagen levels, followed by the exosome-alone group, aligning with reports on the pro-regenerative roles of exosomes derived from biological fluids and blood derivatives (García Coronado et al., 2024; Zhang et al., 2018; Raghav et al., 2021; Shu et al., 2023).

Additionally, it has been documented that exosomes isolated from tissues under hypoxic conditions possess enhanced regenerative capabilities (Cheng et al., 2023). Consistent with this, the manufacturing conditions of ICRP may induce transient hypoxia, thereby boosting the regenerative potential of its derived exosomes.

Although no statistically significant differences in complete wound closure were observed among groups by the end of the study—likely due to natural contraction of the panniculus carnosus muscle—all treatment groups demonstrated markedly faster wound closure during the early healing phase. It is worth to mentioned that due to the limitation of the mice model (contraction of the panniculus carnosus muscle) histological analyses had to be performed during the early phase of treatment to assess whether accelerate wound healing process occurred. This early acceleration was strongly associated with more effective infection control, which plays a pivotal role in enabling proper progression through the wound healing cascade.

Collagen deposition, typically occurring between days 4 and 21 and considered a hallmark of the proliferative and granulation phases (Falanga, 2004; El-Tookhy et al., 2017), appeared earlier in the ICRP-derived treatments. Previous reports indicate that the exosomes isolated from the ICRP stimulate the proliferation and migration of HUVEC, NIH-3T3, and HACAT cell lines (García Coronado et al., 2023), involved in the re-epithelization of wounds.

Re-epithelialization was evident across treated groups and was accompanied by increased epithelial thickness. This was reflected in the higher number of epithelial cells observed in the wound bed (Oswal et al., 2013). Supporting this, exosomes incorporated into hydrogel matrices have been shown to enhance epithelial thickness and accelerate wound repair, as seen with adipose-derived stem cell exosomes (Shafei et al., 2019).

ICRP has previously been shown to modulate inflammatory cytokine expression in cells exposed to LPS-induced stress (Franco-Molina et al., 2005), as well as regulate transcription factors NF-κB and NFATx in the MCF-7 cell line (Mendoza-Gamboa et al., 2008). These findings led us to hypothesize that exosomes isolated from ICRP may exert similar immunomodulatory effects.

Furthermore, is to know that exosomes as carriers have been shown to downregulate key pro-inflammatory cytokines at the wound site, and exosomes derived from hemoderivatives like ICRP, studies with PRP- and MSC-derived exosomes show they accelerate healing by potentially promoting M2 macrophage polarization (Bakadia et al., 2023). It has been pointed out *in silico* that the exosomes from the ICRP stimulate macrophage polarization and angiogenesis (García Coronado et al., 2024).

In our study, serum IL-12p70 levels were significantly reduced by day 21 in mice treated with ICRP or its pellet fraction, consistent with previous data, associated to enhanced tissue repair (García Coronado et al., 2024; Matias et al., 2011; Wang et al., 2013).

TNF-α levels were also significantly lower in pellet-treated mice on days 7 and 14 compared to the positive control. Given that elevated TNF-α impairs healing in diabetic wounds (Landis et al., 2010), its downregulation likely contributed to the accelerated repair observed, as previously reported in third molar surgeries treated with ICRP (Franco-Molina et al., 2016).

MCP-1, a chemokine associated with impaired healing when overexpressed in diabetic wounds (Landis et al., 2010), was maintained at intermediate levels in ICRP and pellet-treated animals—lower than the negative control at day 7, but higher than the positive control. Suggesting a balanced inflammatory response. Since MCP-1 also supports macrophage recruitment (Worsley et al., 2022; Singh et al., 2021), controlled expression may promote wound progression. By day 21, MCP-1 levels had further declined in treated groups, indicating resolution of inflammation and transition to tissue regeneration.

Similarly, IL-6 expression was generally reduced in ICRP and exosome-treated groups compared to the negative control, aligning with prior findings in diabetic wound healing models (García Coronado et al., 2024; Franco-Molina et al., 2005). As elevated IL-6 is characteristic of delayed healing in type 2 diabetic patients (Galicia-Garcia et al., 2020), its downregulation supports the improved regenerative outcomes observed. These results agree with studies showing that platelet-rich fibrin and hyaluronic acid reduce IL-6 while enhancing angiogenesis (Kartika et al., 2021). Correspondingly, angiogenesis was improved in our treated groups, as reflected by the Greenhalgh scoring.

In contrast, IL-10, a cytokine involved in tissue repair and its capacity to inhibit excessive fibrosis, thereby promoting better scar quality (Short et al., 2022; Short et al., 2021), was upregulated on days 7 and 14 in all treated groups. Together, these findings suggest that the modulation of IL-6 and IL-10 by ICRP and its components facilitates resolution of chronic inflammation and promotes tissue regeneration.

It is known that the exosomes preserve modulatory characteristics as its cellular origin. In this case, the ICRP is a complex mixture of substances, including the exosomes secreted by immune system cells, preserving the capacity of modulating the inflammatory microenvironment (García Coronado et al., 2024; García Coronado et al., 2023), accelerating the wound healing process. It could be observed that anti-inflammatory cytokines increased their expression (such as IL-10), probably pointing out towards a M2 macrophage polarization.

The phosphoinositide 3-kinase (PI3K)/AKT signaling cascade plays a central role in intracellular pathways regulating of wound regeneration. Exosomes have been reported to activate the AKT pathway, thereby accelerating tissue regeneration (García Coronado et al., 2024), in part through phosphorylation of FOXO transcription factors and induction of genes associated with cell proliferation and survival (García Coronado et al., 2023; Di-Poï et al., 2002). Likely to exosomes isolated from ICRP exhibited the highest levels of phosphorylated AKT and FOXO, consistent with previous findings (García Coronado et al., 2024). Nevertheless, a high phosphorylation of inhibitory markers of the pathway persists (P21, TSC2), the increase in the presence of AKT and FOXO in phosphorylated state is greater than last ones, implying an activation of the proliferation pathway confirmed with the regeneration of the wound downstream. In addition, it can be corroborated the activation of the pathway downstream due to the production of collagen and organization of the dermal layers in treated group by day 21. These results suggest that ICRP and its components modulate the inflammatory microenvironment in a balanced manner that supports regeneration.

## 5 Conclusion

Exosomes derived from IMMUNEPOTENT CRP enhanced the therapeutic efficacy of gentamicin in diabetic wounds infected with *S. aureus*. By enabling pH-responsive drug release, reducing bacterial burden, and modulating inflammation, these exosomes accelerated wound healing. Altogether, our findings support their dual role as both nanocarriers and bioactive agents, positioning them as a promising strategy for managing chronic infected wounds. Further studies are needed to identify the specific molecular components within ICRP-derived exosomes responsible for their regenerative and immunomodulatory effects. Additionally, evaluating macrophage polarization suggestive of M2 polarization and validating these findings in larger animal models will be essential steps toward clinical translation.

## Data Availability

The original contributions presented in the study are included in the article/Supplementary Material, further inquiries can be directed to the corresponding authors.
