# Supplementary figures and images for: Gentamicin-loaded exosomes from IMMUNEPOTENT CRP enhance healing of infected diabetic wound in mice
Source: Front Pharmacol. 2025 Nov 27;16:1682468. doi: 10.3389/fphar.2025.1682468 (PMC12695595; doi:10.3389/fphar.2025.1682468)

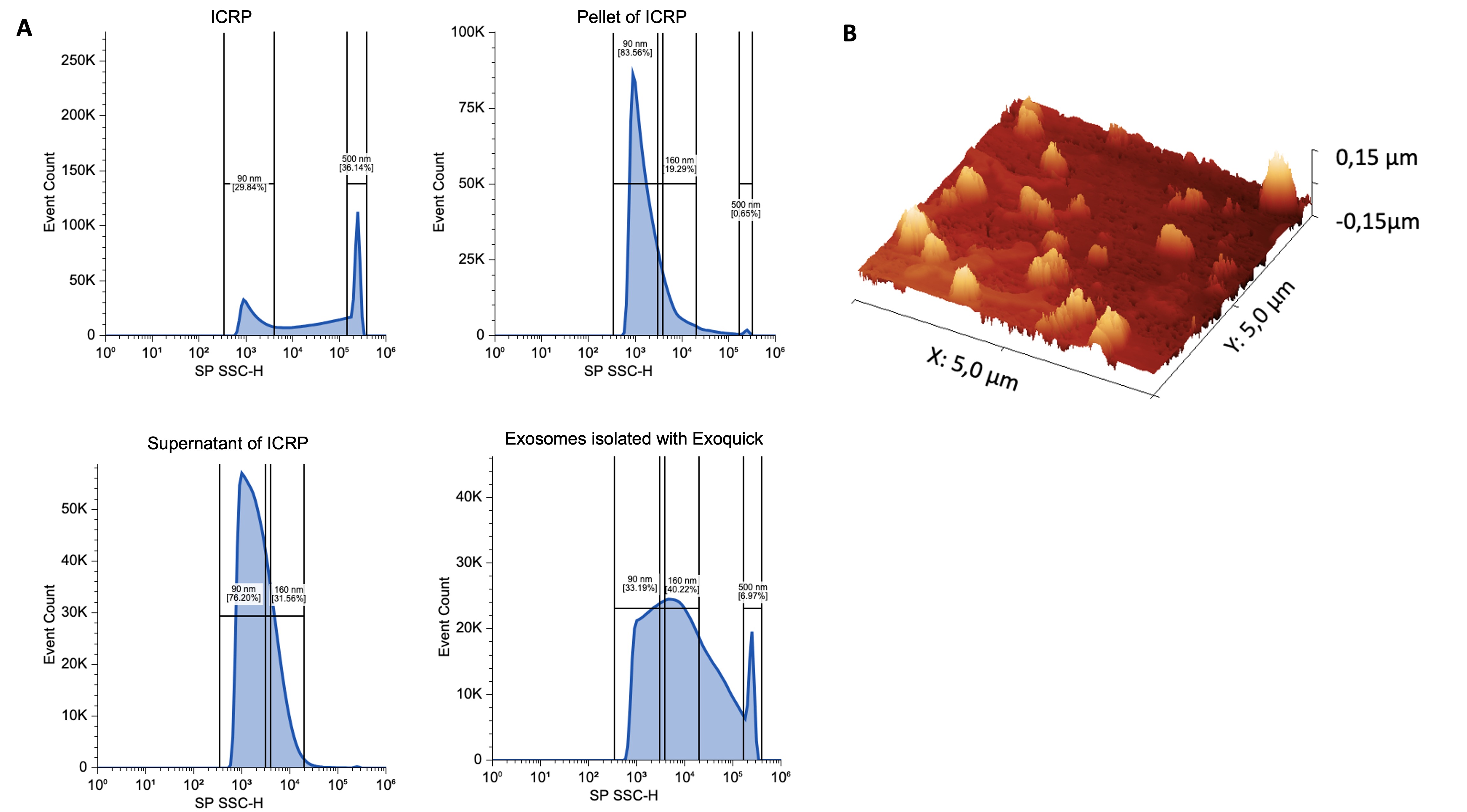

Supplement: Supplementary file 1 [file Image1.jpeg]

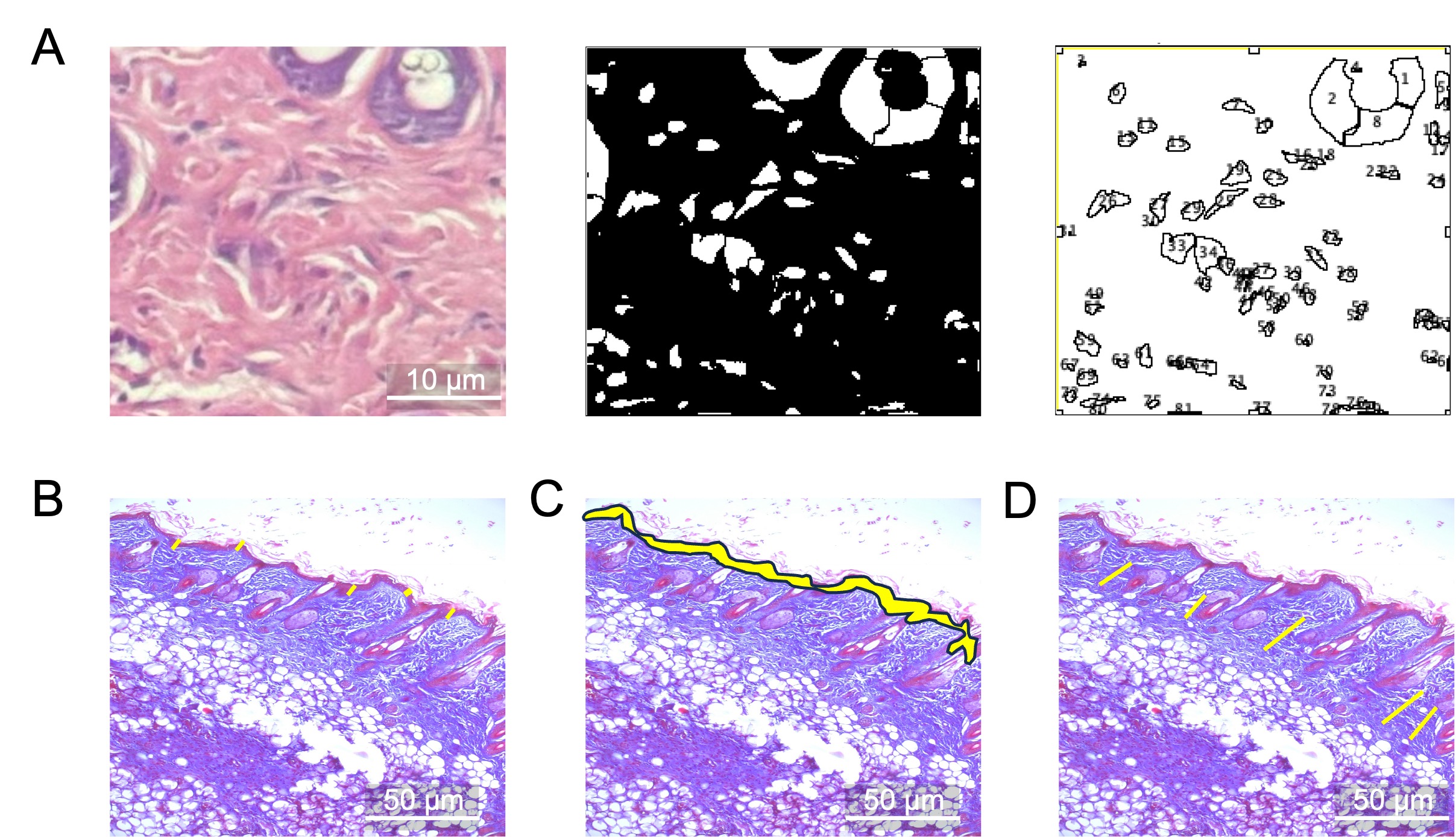

Supplement: Supplementary file 2 [file Image2.jpeg]
